# Renewable energy consumption and economic growth. Causality relationship in Central and Eastern European countries

**DOI:** 10.1371/journal.pone.0202951

**Published:** 2018-10-08

**Authors:** Marius-Corneliu Marinaș, Marin Dinu, Aura-Gabriela Socol, Cristian Socol

**Affiliations:** Department of Economics, The Bucharest University of Economic Studies, Bucharest, Romania; Shandong University of Science and Technology, CHINA

## Abstract

The new European model stipulates the achievement of an inclusive, sustainable and intelligent economic growth. Increasing the share of renewable energy is one of the factors that improve the quality of economic growth, similar to research, development and investment in human capital. In this paper we tested the correlation between economic growth and renewable energy consumption for ten European Union (EU) member states from Central and Eastern Europe (CEE) in the period 1990–2014, using Auto-regressive and Distributed Lag (ARDL) modeling procedure, a technique that captures causal relationships both on a short run and on a long run. The short run perspective reveals the transition towards a new energy paradigm, while the long run approach corresponds to the long-term equilibrium of the analyzed factors. Our results shows that, in the short run, the Gross Domestic Product (GDP) and Renewable Energy Consumption (REC) dynamics are independent in Romania and Bulgaria, while in Hungary, Lithuania and Slovenia an increasing renewable energy consumption improves the economic growth. The hypothesis of bi-directional causality between renewable energy consumption and economic growth is validated in the long run for both the whole group of analyzed countries as well as in the case of seven CEE states which were studied individually. These results allow us to look into the feasibility of the Europe 2020 goals regarding the increase of energy efficiency and to propose public policies to achieve these goals.

## 1. Introduction

The subject of our study is consistent with the objectives set by the European Union within the Europe 2020 Strategy aimed at reducing the primary energy consumption and achieving a consistent share of renewable energy in the final energy consumption. Thus, increasing energy efficiency should lead to lower energy demand per unit of GDP in the economy, a cut of greenhouse gas emissions and the identification of long run solutions to reduce the dependence on traditional energy sources.

The increased energy costs along with the strategic goals undertaken globally in the area of air pollution reduction have contributed to a more elaborated study of the links between renewable energy consumption and the economic growth. In many European economies relying on energy consumption, a trade-off may occur between measures to reduce air pollution and those to increase energy efficiency, on one hand, and the measures aimed at stimulating the economic activity, on the other hand. In case of economies facing a decoupling of growth from energy consumption, this trade-off doesn`t exist and the reforms targeting a structural change in the energy consumption are less expensive to economic activity.

In general, the industrial sector of an economy is more energy intensive compared to service sector therefore the latter stimulates economic growth in the context of a relatively lower energy consumption. Under these circumstances, the more developed EU economies, with a lower proportion of industry will enjoy a reduced energy consumption per GDP unit. According to [1], industry accounted for 19.4% of GDP at the EU level (2015), compared to 26–27% of GDP in Romania, Slovakia, Slovenia and Poland, while the energy intensity was 120 kg of oil equivalent/GDP unit at the EU level, whereas in the CEE states it was about 80% higher. For the economies where economic growth is not influenced by energy consumption, measures to reduce emission of air pollutants by cutting dependency of traditional fuel and increase renewable energy consumption will be quickly implemented in line with the strategic European goals.

The first author that argued about the need to incorporate the physical dimension of output, including the energy in the economic growth theory, was [2]. The 1971 oil shocks triggered an inclusion of energy among the production function along with the traditional factors–labor force and capital stock across the economy, like in the studies [3] and [4]. Today, energy is considered as one of the factors of economic growth and its impact is starting to be quantified—both quantitatively and qualitatively—in the factors of production functions belonging to the two broad categories of models—the Solow model and the AK models [5–8].

Specifically, increased production and renewable energy consumption are already seen as some of the potential endogenous growth engines, similar to research, development and investment in human capital. Economic development based on efficient energy consumption and on a higher share of green energy in the energy mix involves an improved quality of economic growth, making it more sustainable. Moreover, lately, the economic scientific literature is rich in analyses on the sustainability of economic growth in terms of social inclusion and environmental impact–an environment-friendly economic growth fostering also the reduction of inequities. The compatibility between social justice and environmental protection is a fundamental condition of social welfare.

Thus [9] and the Treaty on the European Union stipulate three dimensions of sustainable development—economic, social and environment–underlining that durable development strategies should mainly focus on the structural change of consumption and output in order to boost support for a rational use of natural resources, to diminish the polarization of revenues, and increase economic competitiveness. The authors [10] have argued that economic growth rate represents a sustainable development goal if it is disconnected from energy consumption and from the negative impact on the environment.

Also, the European Commission’s proposal under the 2030 Agenda [11] includes a series of Sustainable Development Goals, with a leading focus on the access to energy primarily due to equality and social justice reasoning. The policies aimed at reducing poverty are also offering easy access to energy along with sustainable chances for development to a range of low-income population [1]. According to [12] accessible and clean energy is vital for a healthy and durable economic development. Thus, the energy has an important role in achieving the sustainable development objective of EU economies [13].

From the methodological point of view, there are recent attempts to include indicators of environmental quality in the overall concept of competitiveness at country level. Thus, The Global Competitivity Report 2014–2015 [14] proposes the adjustment of the Global Competitivity Index by a factor related to social inclusion and another factor related to the protection of the environment -social sustainability adjusted Global Competitivity Index and environmental sustainability Global Competitivity Index.

The idea to adjust the method of quantifying the economic development by including components related to social inclusion and sustainability regarding the environment is reflected in several working documents (i.e the Stiglitz-Sen-Fitoussi Report [15], Europe 2020 Strategy [16], Life Quality Index [17] and the Sustainable Development Goals strategy). In terms of quantifying environmental sustainability main contributions are included in Environmental Performance Index (EPI) [18] and the Ecological Footprint [19], developed by the Global Footprint Network and the Global Adaptation Index. The authors [20] have calculated the emissions of carbon as a measurement tool for human well-being.

The Europe 2020 Agenda sets forth measures aimed at achieving more competitiveness, sustainability and innovation in Europe. To implement such a package of measures and monitor its implementation, the World Economic Forum elaborated the Europe 2020 Competitiveness Index, a composite index based on the dynamics in seven areas: innovation, entrepreneurship, education and research, digitalization, employment, social inclusion and sustainable environment development [21].

The goal of our study is to assess the importance of the renewable energy sector in Central and East European countries compared to the results at EU level and to estimate the compatibility between economic growth and renewable energy consumption in the long run, as well as in the short run. The short run analysis allows us to estimate the CEE economies’ reaction in different stages of the transition of their national energy systems to the set objectives by implementing the European environment protection strategies. The long run analysis will enable us to capture the impact of the energy transition through the assessment of the equilibrium between economic growth and a higher share of clean energy.

The importance and contribution to specialized literature of this study derives from three novelties. Thus we aimed to cover a gap in the specialized literature by analyzing a group of 10 Central and Eastern European economies whose selection was not random but took into account their relatively homogenous economic and social developments over the past seven decades. The 10 economies account for almost 15 percent of the EU’s overall renewable energy production, most of them having better results or closer to the EU target regarding renewable energy’s share in the total energy consumption. We have also overcome the trap of analyzing only the aggregate results by applying an ARDL model to examine the relationship between economic growth and renewable energy consumption on both short- and longer-term for each of the 10 economies. Moreover, we stopped short of explaining the differences between the short-term and long-run coefficients solely in an econometric manner. Thus, we substantiated which are the coordinates of the transition towards a new energy paradigm (on the short-run) in order to match the long-term relationship between the economic growth and renewable energy consumption.

The study/paper structure is in line with the assumed objective and the contributions to the specialized literature.

First, we review the findings of the most relevant studies published in the literature on the subject chosen for our research and we set the main hypotheses on the possible correlation between renewable energy consumption and economic growth. Second, we analyzed two of the key economic development coordinates of CEE countries—economic growth as a result of the process of transition to a functioning market economy and increased energy efficiency as a result of the transition to the predominant consumption of renewable energy. With regard to the energy component, we explained the features of the EU countries (with details on the analysis of the CEE economies) regarding the importance of the renewable energy sector, efforts to reduce emissions of greenhouse gases and evolutions of energy intensity and of the degree of energy dependence. In the next section we tested the hypotheses about the relationship between economic growth and renewable energy consumption in the case of ten EU member economies from Central and Eastern Europe in the period 1990–2014 using the Auto-regressive and Distributed Lag (ARDL) approach, which allows us to capture the causality relations between GDP and REC both on a short run and on a long run (a cointegration relationship). Presentation of results and their interpretation is made in Section 5. The last section proposes several conclusions on the issue studied in the paper.

## 2. Literature review

In the literature there is no agreement on the links between energy consumption and economic growth because the structural features of the analyzed countries, their development stage, econometric methods used and time frame analyzed are different. However, some researchers, such as [22–26] have made a synthesis of the main results of the literature, highlighting that four different hypotheses were tested and confirmed.

The first, called *"non-causality hypothesis"* or *"neutrality hypothesis"* argues that there is no econometrically valid relationship of dependency between energy consumption and production of final goods in an economy. This happens in countries whose real GDP growth relies, to a greater extent, on service sector involving low energy consuming. Therefore, validating this hypothesis implies that policies which seek to reduce energy consumption in order to decrease greenhouse gas pollution effects will not negatively affect domestic output. Namely, the economy can be regarded as being decoupled from the dynamics of energy consumption.

The second is the *"uni-directional causality from economic growth"* or *"conservation hypothesis"* according to which real GDP growth influences the consumption of energy. In this case too, the decisions to decrease the consumption of energy will have only a marginal impact on the dynamics of an economy. *Conservation hypothesis* can be analyzed both in the context in which the economic activity leads to higher consumption of energy, and also in the fact that economic activity leads to lower consumption as a result of constraints on the use of resources and reduced demand for products with high energy consumption.

The next hypothesis is the *"uni-directional causality from energy consumption"* or *"growth hypothesis"* according to which the consumption of energy has a great influence on the economic growth process. If there is a positive relationship between these variables, then the pollution reduction measures will negatively affect domestic output. However, the economic reality has shown that there may be a negative relationship between consumption of energy and real GDP growth that can be interpreted differently depending on the exogenous variable change. Thus, lowering energy consumption positively boosts the domestic output if an economy will be based, to a greater extent, on service sector which are less energy consuming. Similarly, the increased consumption of energy has a negative impact on the GDP, if an economy is based on sectors with high energy intensity and low energy efficiency.

The last hypothesis is the *"bi-directional causality"* or *"feedback hypothesis"* according to which the energy consumption and economic growth are interdependent. Thus, increasing consumption of energy leads to higher real GDP, which in turn positively affects energy consumption nationwide. In this case, environmental policies will generate both consumption and GDP decrease, and the economic stimulus measures will lead to both GDP growth and the increase in energy consumption.

Moreover [25] summarized the results of 48 studies published in prestigious international journals, according to the above four hypothesis. Thus, 29% of researches have confirmed the impact of energy consumption on domestic output, 27% have validated the feedback hypothesis, 23% highlighted a statistically significant impact of the GDP on consumption of energy, while 21% of the studies have not found a relationship between the two variables.

The dependency relationship between REC and real GDP growth has been less studied, because the issue of production and renewable energy consumption has been undertaken only recently as a fundamental objective for their future economic development. In this field, [27] estimated that the macroeconomic efficiency of 45 countries has been improved due to higher renewable energy consumption. The first author who has studied the relationship between renewable energy and domestic output growth was [28,29]. He estimated for G7 economies and for the Organization for Economic Co-operation Development (OECD) economies that the increase of the real GDP per capita has a great impact on the renewable energy consumption per capita. Also [30] validated the feedback hypothesis for 19 developed and developing economies during 1984–2007 and [31] demonstrated, for 20 OECD economies, that there is a positive impact of renewable energy consumption on the long run economic growth, if taking into account the classical factors of potential GDP growth–gross fixed capital formation and employment. The first analysis for the EU member states was conducted by [32] who showed that between 1997 and 2007 there was no statistically significant relationship between economic growth and renewable energy. Other researchers [33] argued that increasing renewable energy production in OECD economies (1980–2007) allowed both accommodating additional energy demand and also the support of the sustainable development objective, but without compromising the potential domestic output. The authors [24,34] have both validated the growth hypothesis and the reverse causality, from the economic growth. Other authors [35] replaced the economic growth with the development level of the 154 economies surveyed to better capture the impact of production from renewable sources. Thus, economic development has led to long run increase in renewable production, but only in the case of the already developed economies. Moreover [36] innovated on the traditional analysis of growth and renewable energy, arguing that the bi-directional causality is validated only if control variables are introduced, such as political stability and capital stock. Other researchers [37] estimated for the G7 economies (1990–2011) that there is a bi-directional causality between real GDP growth and renewable energy consumption. This conclusion was confirmed by [38] for a group of emerging economies and by [39] for 80 economies during the period 1990–2012. The conclusion of those authors is that renewable energy is important for economic growth, which in turn stimulates investment in renewable energy production or consumption from such alternative sources. In these circumstances, government policies can pursue both the economic growth targets and environmental protection. Another relevant research [40] revealed that in OECD economies (1990–2010) there was a positive long run relationship between the real GDP, the share of renewable energy consumption, the investment, the employment rate and the R&D expenditure. Also [41] recommended that EU decision makers should make further progress in reducing non-renewable energy and developing other sources of the energy, because the alternative sources will boost the economic growth. In this field [42] demonstrated, on the basis of nonparametric techniques, used for the first time in this context, the fact that in the case of the emerging economies there is a non-linear M-shaped relationship to a consumption level of 10 terawatts per hour, and then a U-shaped relationship between renewable energy consumption and real GDP growth. In the case of the high income countries, there is also a one-way relationship between those variables, only if the consumption from renewable is less than 50 terawatts per hour.

Another researcher [43] conducted the first meta-analysis of the link between renewable energy consumption and economic growth on the basis of 40 empirical studies concluding that the probability of validating the neutrality hypothesis is greater on the short run than on the long run, while the probability of validating the conservation hypothesis is similarly distributed according to the timeframe. In terms of feedback and growth hypotheses, representing the majority among the results obtained, the relationship between the two variables was validated rather on the long run.

The authors [44] have analyzed the relationship between consumption of renewable energy and economic growth on both short and long run, concluding that while for the short run there is a uni-directional causality link between economic growth and renewable energy consumption, in the long run we may speak about a bi-directional link. Also [45] has empirically estimated the link between CO2 emissions, renewable energy and non-renewable energy and economic growth, concluding that the heightened renewable energy consumption had positively influenced economic growth, while conventional energy had no positive impact on real GDP, especially in developed countries. Other researchers [46] applied ARDL methodology to a sample of 28 states to analyze the short run and long run relationship between energy consumption and economic growth underlining that renewable energy had no impact on economic growth while non-renewable energy had influenced that process.

Moreover [47] used a dynamic panel of equations for 72 states using 1990–2012 data to demonstrate the validity of the feedback hypothesis between revenue and renewable energy consumption and between trade and the increase of the consumption of this type of energy. In another study [48] explained the need to change the analysis paradigm of the effects of an increase of the renewable energy proportion on innovation, while [49] study supported the idea according to which energy innovation has a consistent contribution to air pollution reduction.

The authors [26] reviewed the literature concluding that most studies have validated a feedback hypothesis, which is a support to investments in alternative energy field. Moreover, the two authors have noted the trend of the most recent panel studies is to rank countries studied by similar features on energy and economic development.

The research theme regarding the relationship between economic growth and energy (conventional and renewable) has been studied also for emerging countries. Thus [50] have analyzed this relationship, suggesting the estimation of an Index of Sustainable Economic Welfare for emerging countries. Moreover [51] also proposed a detailed Index for Sustainable Economic Welfare (ISEW), based on which to conduct a comparative analysis of the influence of conventional energy and the renewable energy respectively on economic growth. Their study indicates that the feedback hypothesis cannot be validated in the long run except for sustainable economic growth.

The researchers [52] have analyzed the relationship between renewable energy consumption and economic growth on a sample of 17 emerging countries and have estimated that, while the neutrality hypothesis is empirically supported for 12 out of the 17 states, the growth hypothesis was validated only in Peru, the conservation hypothesis only in Colombia and Thailand and the feedback hypothesis proved valid only for Greece and South Korea.

Nevertheless, there are few empirical studies on the relationship between GDP growth and renewable energy in the new Member States of the European Union. Thus [53] analyzed the relationship between these variables in the new EU countries and concluded that there is a statistically significant impact on domestic output only for Bulgaria, Estonia, Poland and Slovenia. Another study [54] analyzed the group of economies in the Balkans and the Black Sea area, concluding that, in Romania, the hypothesis of the bi-directional causality was validated, while in Greece and Bulgaria only the positive influence of renewable energy consumption on the domestic output was confirmed.

The correlation between Romanian GDP environmental taxes and their impact on the welfare is analyzed in the research paper [55]. A detailed analysis on how it can be fostered increasing importance of renewable energy and hence economic growth in rural economies found in research [56] Consistent practical methods that can be used for high-impact renewable energy on the potential of the European economies can be found in the works [57].

Also [58] have analyzed Romania’s renewable energy potential and explained the ways in which it can be capitalized through investments and public-private partnerships. For Hungary [59] proposed an optimization of the action plan aimed to ensure the use of renewable energy taking into account the energy needs and the potential of this country. The researchers [60] have also suggested for Bulgaria public policy measures that would ensure the implementation of a scenario in which the 2020 and 2030 renewable energy consumption targets are met. Looking at the relationship between renewable and non-renewable energy consumption and economic development, [61] underscored that for the Baltics there was a uni-directional causality from economic development towards energy consumption from renewable sources, validating at a great extent the conservation hypothesis.

By analyzing of the specialized literature on the relationship between economic growth and renewable energy we spotted three weaknesses which we have tried to address through our paper/study. First referred to the absence of well-fundamental criteria to establish the kind of economies which were included in various analyses. Even the label of emerging economies would not solve this situation because such a category includes economies form different continents, with divergent evolutions over the past 20–30 years regarding both their economic growth as well as their strategies in the energy sector. The second is that most studies offer explanations for the whole group (in most cases extremely heterogeneous economies) but not for each of the states included in that group. The third relates to the analysis of the short- and long-term relation between economic growth and energy solely in an econometric manner and in the context of weak/superficial/ economic explanations.

Our paper aims to analyze in detail a homogenous group of 10 CEE economies, all EU Member States, to partially cover the gap we identified in the specialized literature. Although some states in our sample are emerging economies, similar to those in Asia or South America, their economic and social developments over the past seven decades (communist period, transition to market economy, aligning to the EU standards and convergence with the EU) require, when studies, a different approach compared to those emerging economies in the world which did not followed the same trajectory. In contrast with other specialized studies/papers, we aim not only to explain the econometric validity of a certain hypothesis regarding the relationship between economic growth and the renewable energy consumption, but also to underline and substantiate the differences between the short-term and longer-run elasticity in the context of two processes–the transition of the national energetic systems to non-polluting resources and the compatibility of the results in the renewable energy with the EU assumed targets (the 2020 Europe Strategy) as well as with the global ones (the 2030 Agenda). The ARDL model allowed us to analyses the short- and long-term elasticity for each of the 10 CEE states.

## 3. Growth and renewable energy sector. CEE economies in the European context

From the point of view of economic development, there should be a sustainable economic growth that will allow for the recovery of the income gap with the European average, ie an improvement of the long-term energy efficiency in the context of ensuring energy security and reducing conventional energy consumption. In both situations, CEE economies have been faced with a transition process—the economic transition from a planned economy to a functioning market economy, namely the transition of national energy systems to renewable energy. We used the variables included in the Table A (in S1 Archive) to characterize the economic growth and relevant energy features in the CEE countries.

### 3.1 Transition and economic growth of CEE countries

Before 1990, all 10 CEE states included in this paper had a centrally-planned economy, were part of a common trade bloc while all the attempts to economic openness or structural reforms have been sporadic (not generalized). Their transition to market economy started with a common scheme applied for all–the neo-liberal Washington Consensus model with main measures including economic liberalization, privatization and the implementation of structural reforms. If price and trade liberalization proceeded swiftly in the all CEE economies, their progress in the economic governance area, competition, labor market, privatization and restructuring of state-owned enterprises was divergent. Thus, Poland, the Czech Republic, Slovakia, Estonia, Hungary and Slovenia followed a shock-therapy transition which meant accelerated sell-offs and reforms. As a result they initially had to face high social costs followed by a fast macrostabilisation and a relaunch of economic growth. In contrast, other states such as Romania and Bulgaria opted for a gradual approach of the transition process due to the high social impact of such economic changes. Reforms were delayed or postponed, therefore they witnessed a more difficult stabilization of the economy while their economic recovery resumed late. The first transformational recession (1991–1993) was followed by a three-year growth and narrowing economic imbalances. As the Asian economic crisis (1997) and the one in Russia (1998) unfolded, some of the CEE economies (the Czech Republic, Bulgaria, Romania) saw a second recession in their first decade of the transition. If compared to the 1990 level, the 1993 GDP in PPP (constant 2011 in USD) one may see that after the first three years of transition to the market economy, Poland lost only 1% of GDP, the Czech Republic and Slovenia witnessed a 12% percent contraction in their output, while the Baltics posted plunges in their GDP ranging from 32% to 48%. Among the 10 CEE economies analyzed, Poland saw the fastest recovery reaching the initial GDP (of the 1990), in 1994. It was followed by Slovenia in 1995, the Czech Republic in 1996 and Slovakia in 1997, while Romania and Bulgaria needed 13 years to exceed the 1990 GDP level, and Latvia and Lithuania 15 years. To conclude, the first decade of the transition to the market economy reveals divergent evolution of the CEE economies despite their common reform route and relative similar initial conditions. Except for Poland, all the other economies saw successive periods of economic growth and decline in their output (Table 1).

**Table 1:** GDP, PPP (constant 2011 international $) - 1990 = 100%). Source: World Bank, 2018.

	1991	1992	1993	1994	1995	1996	1997	2000	2004	2008	2009	2014
**Bulgaria**	92%	85%	84%	85%	88%	89%	88%	90%	112%	146%	139%	145%
**Czech Republic**	88%	88%	88%	91%	96%	100%	100%	105%	120%	148%	140%	149%
**Estonia**	92%	73%	68%	67%	76%	80%	89%	102%	131%	161%	138%	165%
**Hungary**	89%	86%	85%	88%	89%	89%	92%	103%	122%	134%	125%	134%
**Lithuania**	94%	74%	62%	56%	59%	62%	67%	74%	99%	130%	111%	132%
**Latvia**	87%	59%	53%	54%	53%	55%	59%	68%	92%	120%	103%	115%
**Poland**	93%	95%	99%	104%	111%	118%	126%	144%	162%	199%	204%	236%
**Romania**	87%	79%	81%	84%	90%	93%	89%	89%	113%	147%	137%	147%
**Slovakia**	85%	80%	81%	87%	92%	98%	104%	109%	131%	177%	167%	191%
**Slovenia**	91%	86%	89%	93%	100%	104%	109%	123%	141%	172%	158%	160%

Apart from these transition coordinates, the EU accession criteria were similar for all the 10 CEE economies, especially the one regarding the existence of a functional market economy. The accession negotiations between 1998 and 2000 had ended with eight out of the 10 CEE states joining the EU on May 1, 2004, while the other two (Romania and Bulgaria) entered on January1, 2007. The EU Membership and macroeconomic stability had improved confidence of foreign investors regarding the CEE economies, leading to an all-across-the-board economic growth between 2000 and 2008. The GDP (constant 2011 international $) posted a cumulated growth of 75% in eight years in countries such as Latvia and Lithuania, of over 60% in Bulgaria, Romania and Slovakia and of below 40% in the most developed states in the group–the Czech Republic and Slovenia. As a result, income gaps between the CEE economies have been cut compared to 2000, while the gap versus the EU average fell by almost 15 percentage points to showcase the catching-up economic convergence process.

Despite such positive trends, the vulnerabilities accumulated during the period of economic growth had significantly exposed most CEE economies to the effects of the global economic and financial crisis of 2007–2008. As a consequence, nine CEE economies (exception is Poland) entered recession, with GDP (constant 2011 international $) witnessing a contraction ranging from 15% (in the Baltics) to 5% (the Czech Republic and Bulgaria) in one year only. Fiscal consolidation measures helped them reduce budget, commercial and financial imbalances, triggering recovery and a resumption of the CEE economies convergence to the EU levels.

**Fig 1:**
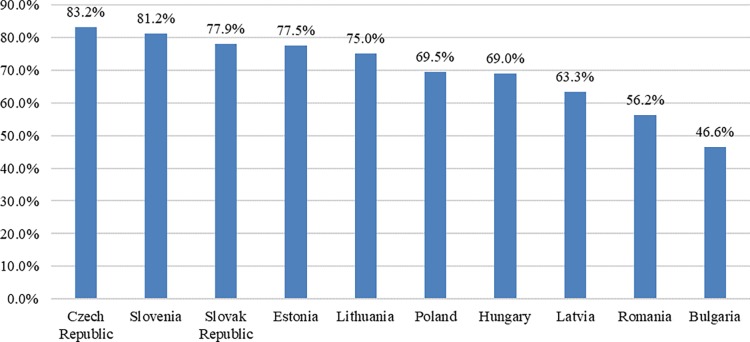
GDP per capita (constant 2011 international $) 2014- EU28 = 100.

In 2014, all CEE states had GDP above their 2009 levels, with Lithuania, Latvia and Poland being the most performant economies with cumulative growth of over 15%. The economic growth contributed to the recovery of income gaps (as GDP per capita) compared to the EU 28 average. Thus, in 2014 the most developed economies were the Czech Republic and Slovenia, with a gap of less than 20 percentage points versus the EU average, while the less developed were Romania and Bulgaria (Fig 1). As compared to the transition’s kick-off moment, the convergence speed was higher in the less developed CEE states and more moderate for the Czech Republic and Slovenia, where GDP per capita was above 70% of the EU average back in 1990.

#### 3.2 Energy features of CEE economies

Regarding the European context, the new paradigm on economic growth model involves structural changes measured by the achievement of several targets set in the Europe 2020 program document: the reduction of carbon dioxide emissions by 20% compared to 1990 levels, the increase in the share of renewable energy consumption in the final energy to 20%, and 20% increase in energy efficiency. Although [12] notes that while there is no common global approach regarding the management of the risks implied by the use of polluting resources, Central and Eastern Europe countries have made great progress to a more sustainable development model in terms of green energy consumption and increased energy efficiency.

Although the trends in renewable energy production are highly sensitive to natural and climatic conditions, Eurostat data show a consistent increase in the amount of renewable energy production in the EU28 by 73% in 2014 compared to 2004. In the European context (with details on 10 CEE countries), the Baltic countries, Romania and Slovenia have set specific targets which are more ambitious than the average EU28 in terms of the share of renewable energy in the final energy consumption (20%), while the Czech Republic, Slovakia and Bulgaria have more lax targets. In the year 2014, Estonia, Romania, Lithuania, Bulgaria and the Czech Republic had achieved their targets committed for 2020, while the EU average gap from the target is 4 percentage points (Table 2).

**Table 2:** Share of renewable energy in gross final energy consumption–CEE countries and EU28 average (%).Source: Eurostat, 2017.

Country / Years	2014	Target Europe 2020
**Latvia**	38.7	40
**Estonia**	26.5	25
**Romania**	24.9	24
**Lithuania**	23.9	23
**Slovenia**	21.9	25
**Bulgaria**	18.0	16
**European Union**	16.0	20
**Czech Republic**	13.4	13
**Slovakia**	11.6	14
**Poland**	11.4	15
**Hungary**	9.5	13

Regarding the distribution by types of renewable energy production there is a focus on biomass & waste (over 50% in all countries analyzed), although there are significant shares in terms of hydropower, especially in Slovenia (44.4%), Romania (26.6%), Slovakia (25.1%) and Bulgaria (21.5%) (Table 3).

**Table 3:** Production of renewable energy and its distribution by types (selected EU countries). Source: Eurostat, 2017.

Country/ Indicators	Primary production (thousand toe)		Share of total, 2014 (%)				
	2004	2014	Solar energy	Biomass & waste	Geothermal energy	Hydro power	Wind energy
**EU28**	113,134	195,814	6.1	63.1	3.2	16.5	11.1
**Bulgaria**	1,009	1,842	6.9	63.6	1.8	21.5	6.2
**Czech Republic**	1,875	3,656	5.4	89.0	0.0	4.5	1.1
**Estonia**	681	1,186	0.0	95.4	0.0	0.2	4.4
**Latvia**	1,837	2,371	0.0	92.3	0.0	7.2	0.5
**Lithuania**	849	1,358	0.5	92.8	0.1	2.5	4.0
**Hungary**	950	2,051	0.5	89.2	6.3	1.3	2.8
**Poland**	4,321	8,054	0.2	89.0	0.3	2.3	8.2
**Romania**	4,594	6,090	2.3	61.9	0.5	26.6	8.8
**Slovenia**	822	1,180	2.8	50.1	2.7	44.4	0.0
**Slovakia**	745	1,441	4.0	70.4	0.5	25.1	0.0

The report [62] contains some interesting developments about the energy sector: an increase in primary production of renewable energy and the amazing development of European investment projects in wind power and solar Photo voltaic in last five years. The strong reliability on renewable energies is also observed in the increased employment in the energy sector. For example, in 2014 year, EU28 registered the second largest share in the number of workers in renewable energy relative to the total population, after Brazil.

A second pillar of the Europe 2020 strategy on sustainability in terms of environmental protection is to reduce the greenhouse gas emissions. The countries analyzed made efforts to meet the targets set in the Europe 2020 strategy (target level—80), the reductions in the emission of greenhouse gas emissions being consistent, compared to the base year (1990).(Table 4)

**Table 4:** Greenhouse gas emissions (1990 = 100).Source: Eurostat, 2017.

Country / Years	2014
**EU28**	77
**Bulgaria**	53
**Czech Republic**	64
**Estonia**	53
**Latvia**	44
**Lithuania**	40
**Hungary**	61
**Poland**	81
**Romania**	36
**Slovenia**	89
**Slovakia**	55

In Romania, according to Eurostat, the greenhouse emissions decreased between 2005 and 2014 by 38% (from 129 million tons of CO2 to 93 million tons of CO2 equivalent—representing 36% of the volume of such emissions for the year 1990), Romania thereby meeting the specific targets assumed [63].

In respect of increasing energy efficiency, the latest Eurostat data indicate primary energy consumption in Romania of 30.8 Mtep in 2014, lower by 0.2 Mtep than in 2013 but still higher than that of many countries from Central and Eastern Europe. (Table 5)

**Table 5:** Primary energy consumption (Million tones of oil equivalent).Source: Eurostat, 2017.

Countries / Years	2014	Target
**European Union (28 countries)**	1507	1483
**Bulgaria**	17,2	16,9
**Czech Republic**	38,6	39,6
**Estonia**	6,6	6,5
**Latvia**	4,4	5,4
**Lithuania**	5,6	6,5
**Hungary**	20,7	24,1
**Poland**	89,1	96,4
**Romania**	30,8	43
**Slovenia**	6,5	7,3
**Slovakia**	15,3	16,4

EU accession of the CEE countries in May 2004 and January 2007 led to increased energy efficiency, their development models being characterized by lower energy intensity. Thus, making a comparison between the situation in 2014 and that in 2007 we can notice that energy consumption per unit of GDP decreased by 12% in the EU28, while in Lithuania it dropped by 31%, in Romania by 26% and in Slovakia by 21%. In Estonia, energy consumption related to GDP increased and there is a marginal reduction in Latvia [64]. (Table 6)

**Table 6:** Energy intensity of the economy (primary energy consumption related to GDP / kg of oil equivalent per 1000 euro).Source: Eurostat, 2017.

Countries/Years	2007	2008	2009	2010	2011	2012	2013	2014
**EU (28 countries)**	138.5	137.6	135.6	137.6	130.3	129.9	128.2	121.5
**Bulgaria**	542.8	509.2	463.9	464.9	490.1	467.8	426.3	445.2
**Czech Republic**	296.2	281.9	277.8	285.7	269.8	270.5	267.9	256.3
**Estonia**	344.4	352.2	372	417.9	390.4	370.3	400.2	390.5
**Latvia**	218.2	217.5	243.9	260.2	231.6	230.9	220.9	215.7
**Lithuania**	294.9	286.6	307.3	242.2	235.8	229.9	209.3	202.5
**Hungary**	258.9	254.8	257.4	261.5	250.1	238.3	225.7	217.7
**Poland**	297.1	288.2	270.6	278.3	265.3	252.8	250.3	233.3
**Romania**	318.8	293	278.3	282.5	285.4	274.4	243	234.7
**Slovenia**	195.1	199.7	197	202.6	201	198.6	195.7	184.5
**Slovakia**	277.3	269	260.7	264.2	250.3	236.3	237.1	220.1

In the current geostrategic context, our analysis on the degree of energy dependence can reveal the vulnerabilities of energy and industrial policies in the Central and Eastern European countries but also the need to increase the share of renewable energy in the energy mix produced by these countries. Thus, the EU as a whole has a high energy dependency, while Lithuania, Hungary and Slovakia are the CEE countries which are the most exposed to a negative shock in energy sector. (Table 7)

**Table 7:** Degree of energy dependency (net imports relative to the necessary gross energy consumption including stocks) (%).Source: Eurostat, 2017.

Countries/Years	2007	2008	2009	2010	2011	2012	2013	2014
**EU28**	52.8	54.5	53.5	52.6	54	53.4	53.1	53.5
**Bulgaria**	50.7	51.7	45.1	39.6	36	36.1	37.7	34.5
**Czech Republic**	25.1	28	27.2	25.6	28	25.3	27.9	30.4
**Estonia**	24.7	24.7	22	13.6	12	17	11.9	8.9
**Latvia**	62.5	58.8	60.4	45.5	59.9	56.4	55.9	40.6
**Lithuania**	61.2	57.8	49.9	81.8	81.7	80.3	78.3	77.9
**Hungary**	61.2	63.2	58.6	58.2	51.8	52.1	52.1	61.7
**Poland**	25.5	30.2	31.6	31.3	33.4	30.6	25.6	28.6
**Romania**	31.7	28	20.3	21.9	21.6	22.7	18.5	17
**Slovenia**	52.5	55.1	48.5	48.6	47.7	51.1	46.9	44.6
**Slovakia**	68.3	64.4	66.5	63.1	64.3	60.2	59.2	60.9

Although it is not a priority objective of this research, a minimum assessment of the dynamics of the EU states’ energy dependency ratio shows that it has continuously picked up since 2000. Given that more than half of the EU Member States imported over 50% of their overall energy consumption (from EU and non-EU countries), we may see a major vulnerability to the eventual sudden fall in the supply or to heightened volatility of energy prices globally. The latest Eurostat data (for 2015) show a low degree of energy dependency (calculated as a proportion of imports in total consumption) in Estonia (7.4%), Romania (17.1%) and Poland (29.3%) and a higher degree in Belgium (84.3%), Ireland (88.7%), Luxemburg, Malta and Cyprus (over 96%) [1].

The new sustainable development strategy at the global level is the 2030 Agenda. The document incorporates 17 Sustainable Development Goals (SDGs) and aims to reduce poverty and to promote inclusive growth compatible with environment protection. Given the theme of our research, the analysis of the latest official statistics demonstrates that the EU countries (especially CEE states) recorded significant progress as regards the fulfilment of the targets under the Sustainable Development Goals—‘affordable and clean energy’, ‘responsible consumption and production’ and ‘climate action’. As an example, in the past 15 years it may be noticed a consistent improvement of energy efficiency and a significant reduction of greenhouse gas emissions, especially due to an increase proportion of renewable energy in the overall energy consumption [1].

The analysis of the recent trends regarding the two major pillars included in the strategy to increase energy efficiency—energy consumption and share of renewable energy in gross final energy consumption—shows that the majority of CEE states register a positive trajectory regarding the observance of their commitments under the Europe 2020 Strategy and the 2030 Agenda. Thus, the energy efficiency should be raised by 20% until 2020 and by at least 27% (with a view to 30%) till 2030, while the share of renewable energy consumption should reach 20% in 2020 and 27% in 2030, according to the 2030 Climate and Energy Policy Framework. The primary energy consumption fell in the CEE economies over the past nine years, except for Poland and Estonia–states whose consumption grew in 2014 versus 2005.

Nevertheless, the comparative analysis of European economies should be regarded with caution, given that the assumed targets under the Europe 2020 Strategy and the 2030 Agenda are different because the initial conditions, including the natural-geographical ones, the structure of renewable energy sources and output capacities are specific. Moreover, according to the [1], the differences between European states derive from both the size of the renewable energy support schemes, as well as from the regulations regarding the introduction with priority of renewable energy in the energy consumption mix. Looking ahead, the EU states will be able to easily meet their commitments regarding “affordable and clean energy" if we take into account the fact that apart from the domestic fiscal stimulus they will benefit from 29 billion euro funds available under the EU Cohesion Policy over 2014 and 2020 also for financing investments to improve energy efficiency, research and development of non-polluting technologies and development of renewable energy output. The rise in renewable energy production should rely on the most advanced and innovative technologies that may ensure significant reduction of greenhouse gas emissions [65].

The effects of an increased share of renewable energy in the output and consumption of EU states have been quantified by the [66]. According to the Report, 77% of the new capacity generated in the EU in 2015 came from renewable energy. Almost a third of the electricity consumption was supported by renewable energy sources, while the increase in renewable energy consumption led to an estimated 10% cut in CO2 emissions and a 2% reduction in primary energy consumption. For the CEE states there is also a significant impact of increased renewable energy consumption on GHG emissions over the past ten years, as well as on fossil fuel consumption and primary energy consumption. Data analysis reveals a strong effect of higher renewable energy consumption growth in CEE countries [66].

**Fig 2:**
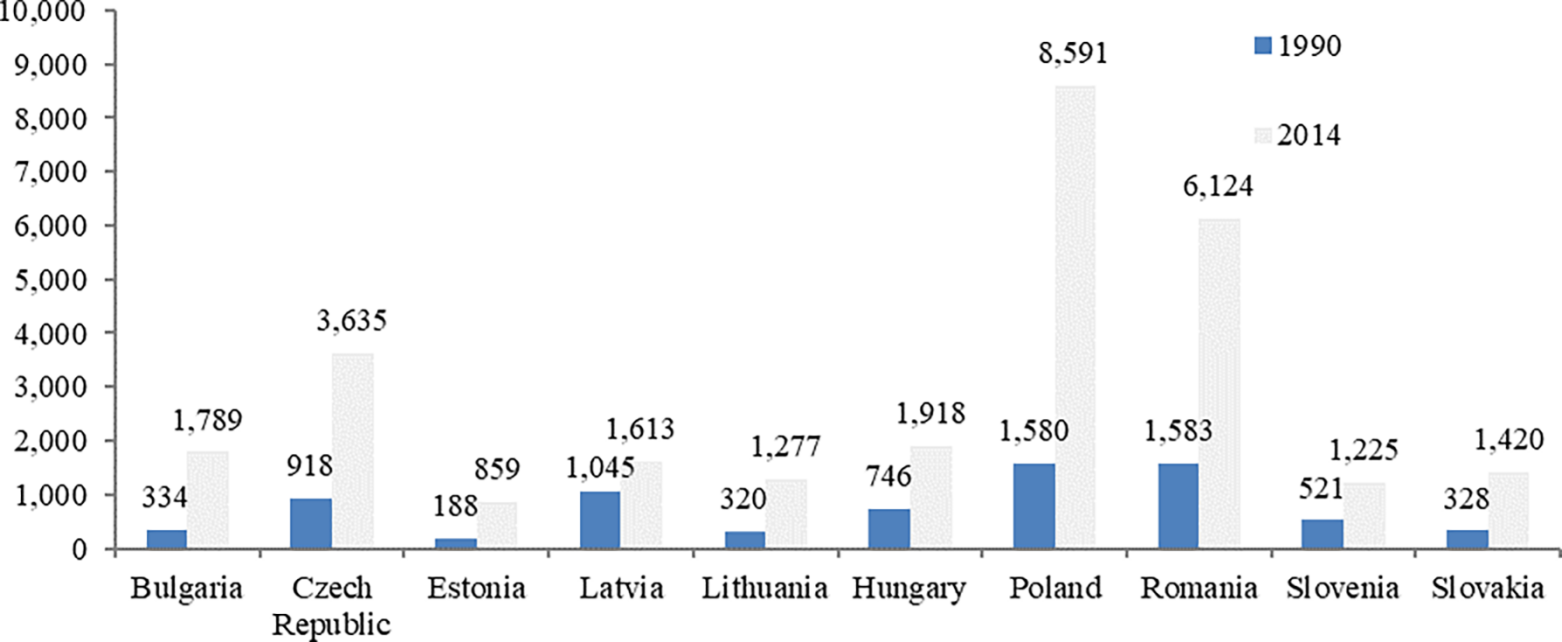
Renewable energies—gross inland consumption (TOE) (1990 versus 2014).

Basically, between 1990 and 2014, renewable energy consumption increased 3.8-fold on average in the countries under consideration, with the largest increases being recorded in Poland and Bulgaria (Fig 2).

Therefore, a rise in the renewable energy output in the CEE states triggered a reduction of air pollution and of energy intensity in the economy, which comes to confirm the importance of the switch towards a sustainable development model from both the economic and energetic perspectives.

## 4. Material and methods

In this study, we used data series of gross domestic product (GDP) expressed in Purchasing Power Parity (PPP) in constant prices of 2011 (international $) and renewable energy consumption (REC), expressed in Thousand tons of oil equivalent (TOE). Annual data from 1990 to 2014 were obtained from the World Bank and from Eurostat (according to Table B in S1 Archive). We used a logarithmic transformation of the two variables, so that the coefficients resulting from panel regressions can be interpreted as elasticities.

The 10 economies from CEE, all EU Member States, have been included in our study based on the following criteria. First, these economies had similar trajectories as regards their economic development–the planned allocation of resources in the economy up to 1990 followed by transition towards a market economy based on the Washington Consensus model, their accession to the EU in 2004 (the Czech Republic, Estonia, Hungary, Lithuania, Latvia, Poland, Slovakia, Slovenia) and in 2007 (Bulgaria and Romania) as a result of meeting certain common conditionalities, i.e. a recovery of development imbalances based on foreign direct investments (mostly from the EU) and on EU funds which were allocated based on the same criteria to all these economies. Secondly, there were some common features of the energy sector–the highest energy consumption to produce a GDP unit, the highest functioning costs of the energy system respectively. Thus, in 2010, the average costs of the energy system were of 12.76% of GDP in the EU compared to 21.3% on average in the 10 CEE states, with values ranging from over 25% of GDP in Latvia and Bulgaria to a minimum level of 17.8% in Slovenia –the most developed economy among the 10 CEE states (EC, 2014). The third is the fact that specialized literature was not interested to analyses this group of economies despite the fact that CEE states enjoy similar economic challenges and context.

The main objective was testing the four hypotheses on the relationship between economic growth and renewable energy consumption in the case of ten EU economies, with a similar level of development and with a common economic and social evolution during the period 1990–2014, despite the divergences arising from the specific features of each economy. Therefore, we intended to identify the type of relationship between the two variables both on the short run, in order to capture the specific dynamics of the economies studied, and especially also on the long run, taking into account the membership to the European economic and social model. In this context, we opted for Auto-regressive and Distributed Lag (ARDL) modeling procedure, which allows capturing causality relationships between GDP and REC both on short run and on the long run, the latter proving a cointegration relationship between the variables. The main reason for selecting such a model is that it is adapted to homogenous economies (such as CEE states), offering a perspective on both the transition process towards a new energetic paradigm for each country, by estimating the short-term relationship, as well as on the impact on the economic development, by estimating the long-term relationship. Beyond the economic arguments, the ARDL model has other advantages regarding econometric estimation accuracy.

Thus, according to [67] the ARDL model, unlike the Engle-Granger or Johansen techniques, can be applied regardless of whether the variables are stationary or become stationary through the first difference, so that the analysis of stationarity becomes a process which is less dependent on the stationarity estimation technique used. The model cannot be used if at least one variable has a higher integration degree, for example I(2). Also, based on the ARDL model, it can obtain an error correction model, which integrates both the short run dynamics, and also the long run equilibrium. In addition, according to [68], the selection of the optimum ARDL model involves automatic correction of the residual serial correlation and of the endogeneity problem. Choosing the ARDL panel is also justified by the recommendations provided in the literature based on the relative length of the period considered and the number of cross sections analyzed. Thus, when there is a relatively smaller group of members compared to the number of years taken into account, then the ARDL approach is used and, otherwise, the Generalized Method of Moments (GMM) is resorted to.

Considering the arguments presented above, in this research we opted for the "Pooled mean group (PMG)" method of estimation, proposed by [69] in a panel of ARDL type. That method is based on a very low degree of heterogeneity between the cross-sections because it leads to the estimation of a long run coefficient available for each member of the panel, while heterogeneity is a feature specific to the short run coefficients, as a result of the dynamics recorded by each economy. In this study, the long run homogeneity of the ten economies results from several common factors, such as the period of transition to a market economy, the process of accession to the European Union and trade and financial links with it, adapting national energy strategy to policies set at EU level and to the objectives set by the Kyoto Protocol. Heterogeneity among the ten economies is valid only on the short run due to the structural features of each of them, which causes a different reaction of economic growth depending on renewable energy consumption. In conclusion, on a short run, it takes into account the differences between economies, while, on a long run, it assumes the existence of identical effects. Therefore, the PMG estimation method it applies if there is a long-run relationship (ie cointegration) between the dependent variable and the independent variables. Once this hypothesis confirmed, the long-run coefficients are estimated based on the ARDL model.

According to [69], the relationship between economic growth (GDP) and renewable energy consumption (REC) can be written as the ARDL type panel, as follows:

$${\Delta GDP}_{it}{=\alpha }_{1i}+\sum_{j=1}^{m-1}\beta_{ij}\Delta {GDP}_{i,t-j}+\sum_{k=0}^{n-1}\gamma_{ik}\Delta {REC}_{i,t-k}+\delta_{1}{GDP}_{i,t-1}+\delta_{2}{REC}_{i,t-1}+\varepsilon_{1i,t}$$ (1)

$${\Delta REC}_{it}{=\alpha }_{2i}+\sum_{j=1}^{m-1}\theta_{ij}\Delta {REC}_{i,t-j}+\sum_{k=0}^{n-1}\mu_{ik}\Delta {GDP}_{i,t-k}+\sigma_{1}{GDP}_{i,t-1}+\sigma_{2}{REC}_{i,t-1}+\varepsilon_{2i,t}$$ (2)

where GDP, REC represent the logarithm of gross domestic product and consumption of renewable energy, Δ is the operator used to calculate the first difference, ε refers to the model error, and α_i_ is the constant specific to each country *i* of the ten CEE economies.

In order to assess the long run relation between the two variables analyzed by the panel we used two test of cointegration—Pedroni and Kao. Both exploit the Engle Granger procedure applied to the residual of the regression. According to this technique the variables are cointegrated if the two are stationary at the first difference I (1) and if the residual stationary at level I (0). Thus, if the residual become stationary at the first difference (I) there is no cointegration relationship between the two variables. The researcher [70] used the residual of the long-run regression to build four statistical tests for cointegration which assume a homogenous autoregressive term (panel, within-dimension tests), and three statistical tests which rely on the heterogeneity of the autoregressive term (group, between-dimension tests). The first three out of four in the first category are non-parametrical tests–first is similar to a long-run V-ratio, the second is a Phillips-Peron (PP) (rho) statistic test, while the third similar with the Phillips-Peron (t) statistic test. The last in the first category is a parametric test similar to Augmented Dickey Fuller (ADF) (t) statistic one. The second category included a PP (rho) statistic test, another one similar to PP (t) and the third featuring an ADF (t) statistic test. Moreover [71] applied the ADF test to the residual of the model comprising 1st order integrated variables (like Pedroni), assuming that the initial regression had homogenous coefficients, fixed effects but without a deterministic trend. Both cointegrated tests of the panel assume a null hypothesis of”no cointegration”, which is rejected if the probability values are below of a certain statistical significance level.

Once the cointegration relationship between the GDP and REC variables confirmed, it can develop the vector error correction model to analyze the short-run relations between the two data series. To select the optimal lag of each variable in the two ARDL equations, we used the Akaike information criterion (AIC) minimization rule, applied for the variables GDP and REC. The Error Correction Term (ECT) used in both models (3) and (4) refers to the residual resulting after applying the long run equilibrium condition between the two variables. According to the Pooled mean group methodology, on the long run the same coefficients result for each cross-section from the group analyzed:

$${\Delta GDP}_{it}{=\alpha }_{1i}+\sum_{j=1}^{m-1}\beta_{ij}\Delta {GDP}_{i,t-j}+\sum_{k=0}^{n-1}\gamma_{ik}\Delta {REC}_{i,t-k}+a{ECT}_{t-1}+e_{1i,t}$$ (3)

$${\Delta REC}_{it}{=\alpha }_{2i}+\sum_{j=1}^{m-1}\theta_{ij}\Delta {REC}_{i,t-j}+\sum_{k=0}^{n-1}\mu_{ik}\Delta {GDP}_{i,t-k}+b{ECT}_{t-1}+e_{2i,t}$$ (4)

The coefficients *a* and *b* represent the speed of adjustment to long-run equilibrium level. If each of these coefficients is in the (-1, 0) range, then the error correction mechanism is stable and ECT helps to adjust the long-run relationship due to the impact of a specific exogenous shock. In the case of positive *a*, *b* coefficients the ECT model leads to the model deviation from the long-run equilibrium so that a certain shock will no longer be neutralized. If those ratios are closer to 0, then the exogenous shock adjustment is performed at low speed, while the closeness to -1 corresponds to a high shock adjustments in one period taken into account (for example, one year in the case of annual data, a quarter for quarterly data etc.).

The last step in testing the assumptions of economic growth and renewable energy consumption is the application of the Granger causality principles on the short and long run, according to the estimated coefficients of Eqs (3) and (4). Thus, null hypothesis (H_0_) *lack of short-run causality* between REC and GDP is confirmed when *γ_ik_* = 0, and the one specific to the relationship between GDP and REC is validated when μ_*ik*_ = 0. Regarding the *long-run Granger causality*, the hypothesis H_0_ is tested depending on the associated coefficient ECT_t-1_, *a* = 0 (Eq 3) and *b* = 0 (Eq 4). In conclusion, there is *a strong Granger causality* between the variables REC and GDP, and between GDP and REC if the null hypotheses (H_0_) *γ_ik_* = *a* = 0 and μ_*ik*_ = b = 0 are rejected.

## 5. Results and discussion

In this section we showed the results of applying the algorithm of panel ARDL model. Thus, first we tested the stationarity of the two data series used to investigate the validity of the ARDL model. Then, we tested the existence of a cointegration relationship between economic growth and renewable energy consumption. Subsequently, we found the optimum number of ARDL model lags, based on minimum of Akaike criterion. The confirmation of the cointegration relationship allowed us to test the error correction models, based on which we obtained the long-run and short-run relationships between variables. Finally, we tested the four hypotheses of the relationship between growth and renewable energy consumption based on the coefficients resulting from the short run equations of the ten economies analyzed. To estimate the results of the ARDL models we used the Eviews 9.5 software.

### 5.1 The testing of data series stationarity and of the cointegration relationship

To test the presence of a unit root we used four tests specific to the analysis of the panel stationarity, such as [72–74], and the Fisher ADF and PP type tests. The first test considers that individual unit root tests (for each cross section) have limited explanatory power, so individual residuals are standardized in order to apply the test of the unit root at the full panel level—*common unit root*. The Breitung test is similar to it, only it does not consider the deterministic trend in the estimation of individual residuals. Im, Pesaran, Shin and Fisher-type tests cancel the restrictive assumption of Levin, Lin and Chu and Breitung and suppose an *individual unit root*. Thus, the first two categories of tests have null hypothesis of the non-stationarity based on a common unit root and the other two involve estimating the null hypothesis based on an individual unit root. In this case, the null hypothesis is accepted if a variable has unit root for all cross-sections analyzed and is rejected if some of them (but not all) have a unit root. If the null hypothesis is rejected at a certain significance threshold (depending on the probability associated with the test), then that variable is stationary. To test the stationarity of the variables included in the model, we used both the variant in which only the *intercept* is included, and also that of including the *intercept and the trend*, using a maximum number of two lags selected by using the Akaike informational criterion (AIC).

The results obtained after testing the unit root were included in Tables 8 and 9. According to them, there is a mix of results on stationary tests used. For example, the inclusion of a trend generally contributes to reject the null hypothesis at the level—I(0). Thus, the variables GDP and REC have the same order of integration I(0) or I(1) when using only the *individual intercept* or different orders of integration for Levin, Lin & Chu and PP—Fisher Chi-square Tests when using Individual intercept and trend. No variable becomes stationary using the second difference, so that the first condition is validated for using the ARDL model.

**Table 8:** Testing GDP stationarity.

GDP	LEVEL				First Difference				Conclusion	
	Individual intercept		Individual intercept and trend		Individual intercept		Individual intercept and trend		Individual intercept	Individual intercept and trend
	Statistic	Prob.	Statistic	Prob.	Statistic	Prob.	Statistic	Prob.		
Levin, Lin & Chu t* (common unit root process)	-2.61589	0.0044	-0.46856	0.3197	-7.8040	<0.0001	-3.7882	0.0001	I(0)	I(1)
**Breitung t-stat (common unit root process)**	-	-	-1.01067	0.1561	-	-	-1.9536	0.0254	-	I(1)
**Im, Pesaran and Shin W-stat (individual unit root process**	0.8105	0.7912	-2.63633	0.0042	-8.6614	<0.0001	-4.7888	<0.0001	I(1)	I(0)
**ADF—Fisher Chi-square (individual unit root process**	11.3745	0.9359	42.6776	0.0023	106.70	<0.0001	56.965	<0.0001	I(1)	I(0)
**PP—Fisher Chi-square (individual unit root process**	2.42873	1	49.2489	0.0003	100.25	<0.0001	72.575	<0.0001	I(1)	I(0)

**Table 9:** Testing REC stationarity.

REC	LEVEL				First Difference				Conclusion	
	Individual intercept		Individual intercept and trend		Individual intercept		Individual intercept and trend		Individual intercept	Individual intercept and trend
	Statistic	Prob.	Statistic	Prob.	Statistic	Prob.	Statistic	Prob.		
Levin, Lin & Chu t* (common unit root process)	-3.458	0.0003	-3.3239	0.0004	-9.8916	<0.0001	-4.2439	<0.0001	I(0)	I(0)
**Breitung t-stat (common unit root process)**	-	-	-1.0188	0.1542	-	-	-7.7836	<0.0001	-	I(1)
**Im, Pesaran and Shin W-stat (individual unit root process**	0.205	0.5815	-4.1467	<0.0001	-13.015	<0.0001	-10.424	<0.0001	I(1)	I(0)
**ADF—Fisher Chi-square (individual unit root process**	24.064	0.2396	53.4156	0.0001	162.731	<0.0001	118.425	<0.0001	I(1)	I(0)
**PP—Fisher Chi-square (individual unit root process**	10.144	0.9655	24.9271	0.2042	163.531	<0.0001	134.942	<0.0001	I(1)	I(1)

Regarding the tests of cointegration relationship, following individual unit root tests for variables with an Individual intercept (according to Tables 8 and 9) it results that both GDP and REC variables become stationary with the first difference. Therefore Pedroni and Kao cointegration tests may be applied to identify the long-run relationships between economic growth and renewable energy consumption, between consumption and growth respectively.

In line with the results included in Table 10, the Kao test rejects the null hypothesis H_0_ of “no cointegration” for both models at the 1% statistical significance level. The Pedroni test finds the existence of cointegration between GDP and REC, respectively REC and GDP, for 4 out of 7 statistical tests at 5% percent threshold and for 5 tests if the statistical significance level is 10%. The rho statistics for Panel and Group are the only confirming the lack of cointegration in the case of the two models. A long-run relationship between economic growth and renewable energy consumption (irrespective of their order in the model) had been confirmed also by the Fisher-Johansen combined test of panel cointegration. Moreover [75] used Johansen cointegration test [76] t and Fischer [77] recommendation to combine individual tests to achieve a statistic test for the full panel (overall statistic test). To test the null hypothesis of “no cointegration” the likelihood ratio trace statistics and maximum eigenvalue statistics were used. Johansen Fisher test confirms the existence of a cointegration relationship between the analyzed variables at the 1% significance level, according to the probabilities that use the asymptotic Chi-square distribution (Table 11).

**Table 10:** Panel cointegration tests–Pedroni and Kao.

		Cointegration between GDP and REC	Cointegration between REC and GDP
**Pedroni test**	Panel v-Statistic	1.365731*	3.454539***
	Panel rho-Statistic	-1.184897	-0.419110
	Panel PP-Statistic	-3.964512***	-1.962425**
	Panel ADF-Statistic	-2.823269***	-4.088382***
	Group rho-Statistic	-0.087630	0.611567
	Group PP-Statistic	-3.611839***	-1.540912*
	Group ADF-Statistic	-1.736454**	-4.153948***
**Kao test**	ADF Statistic	-3.718325***	-5.017859***

Note

***) 1% significance level

**) 5% significance level

*) 10% significance level

**Table 11:** Panel cointegration test–Johansen Fisher.

Number of cointegration relations	Fisher statistics (trace test)		Fisher statistics (maximum eigenvalue test)	
	Value	Probability	Value	Probability
**None**	121.7	<0.0001	102.0	<0.0001
**At most 1**	47.05	0.0006	47.05	0.0006

Next, we have chosen an optimal lag specific of the ARDL model, according with the minimum levels of the Akaike criterion, obtained with the econometric Eviews software. Thus, the minimum value of the informational criterion specific to Eq 3 was recorded for the ARDL model (2,1), which corresponds to j = 1 in the case of the first difference of GDP, and a k = 0 corresponding to the first differences of REC. In the case of the Eq 4, the optimal lag was 1 both for GDP and also for the variable REC, which corresponds to a ARDL model (1,1).

### 5.2 The estimate of the error correction model, hypothesis test and interpretation of results

The short-run dynamics of the model variables lead, according to the PMG estimator, to different values for each of the ten economies analyzed, in the context in which the long-run relationship is homogenous to the economies in the group. In Table 12 we included the results of applying the PMG estimator for the error correction models described by Eqs (3) and (4).

**Table 12:** Error correction model.

	Dependent variable					
	ΔGDP_it_			ΔREC_it_		
	Coefficient	t-Statistic	P value	Coefficient	t-Statistic	P value
**Long Run Equation**						
**REC**_**it**_	0.661538***	11.08643	<0.001			
**GDP**_**it**_				0.319400**	2.450713	0.0150
**Short Run Equation**						
**ECT**_**t-1**_ **(first model)**	-0.088318**	-2.383885	0.0180			
**ECT**_**t-1**_ **(second model)**				-0.120278***	-3.634241	0.0003
**ΔGDP**_**i,t-1**_	0.376585***	9.087468	<0.001			
**ΔREC**_**it**_	-0.054177***	-3.459625	0.0007			
**ΔGDP**_**it**_				0.274599	1.403972	0.1617
**Constant (first model)**	1.787422**	2.387080	0.0179			
**Constant (second model)**				-0.047213**	-2.009250	0.0457

Note

***) 1% significance threshold

**) 5% significance threshold

Thus, if renewable energy consumption increases by 1% at the CEE countries level, then GDP will increase by 0.66%, which is proof of the positive influence exerted on economic growth by the European and national policies of environmental protection. Also, a 1% increase in GDP leads to growing interest towards alternative energy sources, this being reflected in a higher growth rate of the REC by 0.32%. The two models are stable because the speed adjustment coefficients of exogenous shocks are different from 0 and negative. Thus, the long-run economic equilibrium between GDP and REC is restored after about 12 years, while the equilibrium corresponding to the long-run relationship between REC and GDP is achieved after about 8 years.

We continued with the implementation of the methodology by testing the Granger causality (Table 13). According to Eq (5) F statistics corresponding to the strong Granger causality was calculated by comparing SSR (Sum squared residuals) specific to the unrestricted model with the SSR related to the restricted model obtained by validating the null hypotheses H_0_, ie *γ_ik_* = *a* = 0, and μ_*ik*_ = b = 0 respectively.

$$Fstatistic=\frac{{(SSR}_{Restricted}-{SSR}_{Unrestricted})/no.of.restrictions}{{SSR}_{Unrestricted}/(no.of.observations-no.of.regressors)}$$ (5)

**Table 13:** Granger causality.

	Hypothesis H0 (no Granger causality)		t statistic/F statistic*		Probability of rejecting H0	
	(3)	(4)	(3)	(4)	(3)	(4)
**Short run Granger causality**	*γ_ik_* = 0	*μ*_*ik*_ = 0	-3.459625	1.403972	99.9993%	83.83%*
**Long run Granger causality**	a = 0	b = 0	-2.383885	-3.634241	99.982%	99.9997%
**Strong Granger causality**	*γ_ik_* = *a* = 0	*μ*_*ik*_ = b = 0	48.91216766*	16.5968606*	99.9999%	99.9994%

Note

*) Null hypothesis is confirmed

The value of the F statistic corresponding to model (3) was 48.91216766 and the one for model (4) was 16.5968606. Regarding the Granger causality between the renewable energy consumption and economic growth, our results showed that the long-run relationship is confirmed with a probability close to 100% and the short-run relationship is invalidated. Thus, in the case of the group of the Central and Eastern European countries a bidirectional causality between GDP and REC is confirmed on the long run, which validates the *feedback hypothesis*.

After applying the PMG estimation method for the two models (3) and (4), the result is a homogenous long-run relationships between GDP and REC and between REC and GDP, as well as heterogeneous short-run relationships for each economy of the panel, based on which the speed of shock adjustment and the Granger causality both on the short run and long run could be analyzed.

Table 14 includes the results of testing the *growth hypothesis* (ie the unidirectional causality relationship from renewable energy consumption to economic growth). This hypothesis is validated in the short-run for seven economies–the Czech Republic, Estonia, Hungary, Lithuania, Poland, Slovakia and Slovenia, in whose case the dynamics of renewable energy consumption trigger (in Granger terms) a mild reduction of the economic growth rate. The strategies to stimulate renewable energy production generate certain costs to adapt the national energy system taking into account the investments needed to expand the infrastructure and the intermittent character of such forms of energy. Therefore, the transition towards a higher share of renewable energy may trigger in the short run higher average production cost and a decrease of energy efficiency that will negatively affect the economic growth rate. The short run results are in line with estimates by [5], [46], [78] and [79].

**Table 14:** Growth hypothesis.

	Adjustment Speed	t-Statistic	P value	REC→ GDP (long run)*	ΔREC	t-Statistic	P value	REC→ GDP (short run)
**Bulgaria**	-0.068733	-46.26887	<0.0001	YES	-0.005456	-1.581587	0.2119	NO
**Czech Republic**	0.048573	10.60973	0.0018	NO***	-0.036444	-8.532375	0.0034	YES*
**Estonia**	-0.20070	-65.35233	<0.0001	YES	-0.148625	-23.91951	0.0002	YES*
**Hungary**	0.064224	34.24847	0.0001	NO***	-0.089624	-17.12028	0.0004	YES*
**Latvia**	-0.161265	-31.58744	0.0001	YES	-0.029022	-0.497119	0.6533	NO
**Lithuania**	-0.314513	-104.8871	<0.0001	YES	-0.101462	-5.231563	0.0136	YES**
**Poland**	-0.029730	-43.58588	<0.0001	YES	-0.014728	-43.53131	<0.0001	YES*
**Romania**	-0.005502	-1.651945	0.1971	NO	0.003407	0.765820	0.4995	NO
**Slovakia**	-0.129814	-45.04786	<0.0001	YES	-0.033460	-17.57011	0.0004	YES*
**Slovenia**	-0.085708	-16.41912	0.0005	YES	-0.086361	-23.21263	0.0002	YES*

*) 1% significance level

**) 5% significance level

***) The model is not stable because the coefficient corresponding to adjustment speed is positive and does not allow neutralizing an exogenous shock

In the case of the poorest three economies included in the sample (Bulgaria, Romania and Latvia), real GDP dynamics are not influenced by the change in renewable energy consumption, indicating that in the short run economic growth is decoupled from energy consumption from renewable sources. On the long run, the *growth hypothesis* is rejected in Romania, Czech Republic and Hungary, even if the Granger causality relation is valid for the latter two economies. However, in their case, the error correction model ensures no exogenous shock absorption for ensuring the long run equilibrium. Baltic economies have the highest shock adjustment speed, which is characteristic to smaller economies that have greater economic flexibility. Therefore, in most CEE economies, renewable energy consumption is a useful variable to better forecast the long-run potential output. This means that investments made to increase production and consumption of renewable energy are able to generate a better forecast of the GDP trend.

Table 15 includes the results of the *conservation hypothesis* test. This hypothesis was validated on the short run only in four of the ten economies—Hungary, Lithuania, Slovenia and Latvia. In their case, the forecast of the REC is improved after analyzing the dynamics of GDP in previous years. On the long run, the Granger causality relationship between the two variables is econometrically valid for nine of the ten economies surveyed (except Hungary), highlighting that the economic growth process generates growing demand for energy from renewable resources, which are less polluting. In other words, the forecast of the future values of renewable energy consumption and greenhouse gas emissions will be influenced by the current potential economic growth. According to the coefficients associated with the speeds of shock adjustment, Latvia will reach the long run equilibrium in around three years, while in Poland and Romania the shock neutralization occurs in about 5 years.

**Table 15:** Conservation hypothesis.

	Adjustment speed	t-Statistic	P value	GDP→ REC (long run)*	ΔGDP	t-Statistic	P value	GDP→ REC (short run)
**Bulgaria**	-0.045309	-12.31525	0.0012	YES	0.380703	1.049445	0.3711	NO
**Czech Republic**	-0.037931	-7.294966	0.0053	YES	0.484034	1.366623	0.2652	NO
**Estonia**	-0.139940	-18.23411	0.0004	YES	0.072487	0.392226	0.7211	NO
**Hungary**	-0.000258	-0.137729	0.8992	NO	-0.251585	-2.380758	0.0976	YES**
**Latvia**	-0.344893	-12.27851	0.0012	YES	0.142223	7.623935	0.0047	YES*
**Lithuania**	-0.042297	-15.66950	0.0006	YES	-0.111326	-2.829122	0.0662	YES**
**Poland**	-0.215300	-22.39368	0.0002	YES	1.672034	0.946122	0.4139	NO
**Romania**	-0.187279	-37.95831	<0.0001	YES	0.262914	1.442962	0.2447	NO
**Slovakia**	-0.099077	-14.60026	0.0007	YES	0.698116	1.760152	0.1766	NO
**Slovenia**	-0.090500	-9.516317	0.0025	YES	-0.603613	-2.572871	0.0823	YES**

*) at 1% significance level

**) at 10% % significance level

After centralizing the results obtained in the Tables 14 and 15, it results a grouping of the ten panel economies according to the four hypotheses on the relationship between economic growth and energy consumption (Table 16).

**Table 16:** The four research hypotheses.

	SHORT RUN	LONG RUN
**Bulgaria**	Neutrality hypothesis	Feedback hypothesis
**Czech Republic**	Growth hypothesis	Conservation hypothesis
**Estonia**	Growth hypothesis	Feedback hypothesis
**Hungary**	Feedback hypothesis	Neutrality hypothesis
**Latvia**	Conservation hypothesis	Feedback hypothesis
**Lithuania**	Feedback hypothesis	Feedback hypothesis
**Poland**	Growth hypothesis	Feedback hypothesis
**Romania**	Neutrality hypothesis	Conservation hypothesis
**Slovakia**	Growth hypothesis	Feedback hypothesis
**Slovenia**	Feedback hypothesis	Feedback hypothesis

The results obtained for the group of 10 emerging economies in the Central and East Europe (CEE), are, for most, consistent with the conclusions revealed by other studies focusing on emerging economies, such as [38] and [39], given the empirical estimation of the long-term bidirectional relationship between the consumption of renewable energy and economic growth. Also, [26] and [43] used meta-analysis to conclude that most studies have estimated the validity of the Feedback hypothesis. The specialized literature has so far few references regarding the CEE countries or the economies in this group. Thus, our results may appear in contrast those of [53] because the later validate the growth hypothesis only for four out of seven economies studied—Bulgaria, Estonia, Poland and Slovenia–countries where our study estimated and validated the hypothesis from growth to renewable energy consumption. Nevertheless, in case of Hungary and the Czech Republic the same hypothesis, in line with our long-term estimates, had been confirmed. The 2016 study used the same model (ARDL), but the period it analyzed was trimmed down by 5 years compered to our study. The economic recovery of these economies starting with 2010 had led to a significant increase in renewable energy consumption (+27% in four years) in CEE states, which validate the directional relationship from economic growth to renewable energy consumption.

In line with our results presented in Table 16, there are two major differences between the short run and the long run particularities related to the relationship between growth and renewable energy.

*The first difference refers to the fact that* CEE economies are more homogenous in the long run because the goal is the same–economic development based on a higher share of the output and consumption of clean energy -, but more heterogeneous in the short run, because the transition to the final goal has different effects due to certain gaps such as the structure of renewable energy, the assumed targets and the investments needed to integrate renewable energy into the national energy systems. Thus, on the long run the economic growth process of nine CEE countries (except Hungary) has a positive influence on renewable energy consumption. Moreover, in case of seven CEE states (except the Czech Republic, Romania and Hungary), the strategy to develop renewable energy sources within the energy mix aligned to the Europe 2020 targets has a positive impact in the long run on economic growth because of the positive effects of the accumulation of production factors, energy efficiency at micro level and of green energy technologies. Therefore, for the majority of economies analyzed the feedback hypothesis is validated and confirmed also at the level of the group of 10 CEE economies (see Table 12), which contributes to the design of development models based on renewable energy. In the short run, the lack of causality (in Granger terms) between GDP dynamics and the dynamics of renewable energy consumption for six out of the 10 economies (exceptions include Hungary, Latvia, Lithuania and Slovenia) largely explains the differences between the hypotheses estimated for CEE states. The absence of the causality relation may be attributed to the difficulties of the national energy systems in managing the intermittent and unpredictable character of renewable energy sources. Moreover, in the absence of investments to stockpile energy and expand the network for taking over the renewable energy in the national energy systems, there will be no short run reaction of renewable energy consumption to the swings in economic activity. In the case of the poorer EU economies (Bulgaria and Romania), none of the causalities relations between the analyzed variables was validated, which is proof of both insufficient investments and low demand for the energy resulted from non-polluting, though more expensive, sources. According to the [80], Bulgaria and Romania need the most significant investments in the energy sector, a conclusion revealed also by the increase in the costs of functioning of the energy system by approximately 5% of GDP over 2010–2030, compared to an EU average of 1.3% of GDP.

*The second difference* focuses on the nature of the causality relationship between the analyzed variables. Thus, in the long run, hypotheses such as growth, conservatism or feedback have been econometrically validated in the context of a positive link between economic growth and renewable energy consumption, while in the short run both negative causality relations between the dynamics of the respective variables and the absence of causality between these two variables were estimated. The transition towards non-polluting energy sources may generate costs for economic agents in the short run, such as higher taxes for CO2 emissions, tighter rules for those areas or higher prices of renewable energy that will negatively affect economic activity, as also explained by [81]. In addition, the decision to boost the public financial resources to support such investments, the supply and demand of renewable energy, may trigger a crowding-out effect on other productive investments in the economy, with a negative impact on short-term growth prospects.

## 6. Conclusions and recommendations

This research is the first which includes only those ten CEE countries that followed a common economic and social pattern in the past six-seven decades–the communist period, transition to market economy, post-transition and economic integration in the context of EU accession. We have selected this cluster of economies also due to the common characteristics of their energy profiles, such as the highest energy intensity compared to the EU average both at the start of the economic transition and in the present. Moreover, all ten CEE economies report functioning costs of their national energy systems above the EU average due to an inefficient or obsolete infrastructure.

The issue of renewable energy consumption is important for each country of the panel not only due the lower energy efficiency in the early 1990s, but also to the need for harmonization both national objectives (such as catching up process to reach the average EU level of development) as well as some of the European Union, such as air pollution reduction. Another novelty in the study is the fact that the results of panel ARDL model were explained for each cross section, allowing for the test of the four hypotheses of the relationship between energy consumption and growth at the individual level, both on the short and the long run.

Similar to the transition process of these economies, which proved expensive in the short run (in terms of economic growth, employment, revenues, etc.), but beneficial in the long run, with EU accession and integration, the energy transition towards renewable sources may negatively affect CEE states in the short run due to the structural characteristics of their national energy systems, but it will be beneficial in the long run, in line with the targets set by the Europe 2020 Strategy and the 2030 Agenda. Our results confirm the normal effects of the structural transformations specific to the change in the energy paradigm at EU level, but also the fact that, on the long run, CEE economies will be able to successfully accommodate the costs of this transition to make it fit with the economic growth process. Therefore, the development model of these states will rely on both an increase in the efficiency of using green energy and higher consumption of renewable energy resources at the expense of conventional ones.

Testing the relationship between economic growth and renewable energy consumption for ten CEE countries in the period 1990–2014 showed that, on the short run, the GDP and REC dynamics are independent in Romania and Bulgaria, while in Hungary, Lithuania and Slovenia the measures to increase the renewable energy consumption improve the forecast of GDP, which in turn improve the quality of forecasting the energy consumption—*feedback hypothesis*. In this case, there is no contradiction on the short run between the measures to reduce air pollution and those stimulating economic recovery. Moreover, the *feedback hypothesis* is confirmed on the long run for seven of the economies, proof of concordance between the potential economic growth and the measures to encourage the renewable energy consumption in the national economic development models. In the cases of the Czech Republic and Romania, the measures with economic impact on the long run will affect the renewable energy consumption, with positive effects on reducing air pollution, while in Hungary no causality between potential growth and long run energy renewable consumption is valid.

Some of the CEE countries have exceeded the target set on renewable energy, promoting programs with a major impact in this regard: one of the most effective/supporting incentive schemes through green certificates, support schemes through regulated prices set for each technology—targeted at small producers of energy especially for biomass/biogas, upgrading and creating new production capacities for electricity and heat through investments in hydro, biomass, wind and biofuel sectors. Most of these programs are financed from structural and cohesion funds from the EU and co-financed from the government budget.

Achieving the effective compromise between rapid economic growth and environmental protection means thinking of a different model of growth, as it is foreseen in Europe 2020 strategy and 2030 Agenda—a smart, sustainable and inclusive economic growth. Last but not least, the transition of the CEE countries from the stage of development of the economy based on efficiency to the economy based on innovation depends on increasing the share of renewable energy and increased energy efficiency.

Regarding policy recommendations, the diagnosis analysis of the energy sector in terms of renewable energy confirms that government policies aimed at increasing energy efficiency and promoting the production and consumption of green energy must pursue two main objectives: stimulating economic sectors with low greenhouse gas emissions and reducing such emissions especially in the energy and transport sectors. In addition to the information and training component, the Central and Eastern European countries have implemented programs of concrete environmental protection measures–car fleet renewal schemes, granting subsidies for the purchase of electric/hybrid cars, along with creating a consistent infrastructure of recharging stations for electric cars, national programs for reforestation of degraded land and achieving forest belts, programs for consolidation / expansion of environmental and water supply and sewerage infrastructure and also programs for the construction of modern treatment plants for wastewater / integrated waste management.

To stimulate the production and consumption of energy from renewable sources, we believe that the 2020–2030 National Reform Plans for CEE countries must provide three strategic goals: increasing the effectiveness of support schemes for renewable energy, increasing investments in infrastructure through which to exploit renewable energy sources, and encouraging the development of renewable energy production from less exploited sources. Increasing energy efficiency can be achieved sustainably in the ten CEE countries by promoting State aid schemes for high efficiency cogeneration (granting financial incentives to producers of electricity and heat for high efficiency cogeneration plants with savings of at least 10% compared to separate production), programs on the installation of heating systems using renewable energy (such as Green House) and programs for thermal rehabilitation of residential buildings financed from EU funds.

The focus on renewable energy brings many benefits with economic and social impact, in addition to environmental ones. Reducing dependence on energy imports (an important benefit to the CEE countries, especially in the new geostrategic conditions), less polluting greenhouse gases emissions, increasing employment in the energy sectors, and also increasing intensity of growth by using green technologies (and therefore enhancing innovation in industry) are just some of them.

## Supporting information

S1 Archive Supporting information. **7z** S1 Archive contains two tables; **Table A and Table B.**
(7Z) Click here for additional data file.

## References

[1] Eurostat. Sustainable development in the European Union. Monitoring report on progress towards the Sustainable Development Goals in an EU Context 2017.

[2] Georgescu-Roegen N. The Entropy Law and the Economic Process; Harvard University Press: Cambridge, 1971.

[3] Tintner G, Deutsch E, Rieder R. A Production Function for Austria Emphasizing Energy. De Economist 1977; 125(1): 75–94. [View Article] [Google Scholar]

[4] Berndt ER, Wood DO. Engineering and Econometric Interpretations of Energy-Capital Complementarity. American Economic Review 1979; 69(3): 342–354. [View Article] [Google Scholar]

[5] Bhattacharya M, Paramati SR, Ozturk I, Bhattacharya S. The effect of renewable energy consumption on economic growth: evidence from top 38 countries. Applied Energy 2016; 162: 733–741. [View Article] [Google Scholar]

[6] Destek MA. Renewable energy consumption and economic growth in newly industrialized countries: evidence from asymmetric causality test. Renewable Energy 2016; 95: 478–484. [View Article] [Google Scholar]

[7] Shahbaz M, Rasool G, Ahmed K, Mahalik MK. Considering the effect of biomass energy consumption on economic growth: fresh evidence from BRICS region. Renewable and Sustainable Energy Reviews 2016; 60: 1442–1450. [View Article] [Google Scholar]

[8] Dvorak P, Martinat S, der Horst DV, Frantald B, Turečkova K. Renewable energy investment and job creation: a cross-sectoral assessment for the Czech Republic with reference to EU benchmark. Renewable and Sustainable Energy Reviews 2017; 69: 360–368. [View Article] [Google Scholar]

[9] United Nations—Department of Economic and Social Affairs. World Economic and Social Survey 2013—Sustainable Development Challenges E/2013/50/Rev. 1: ST/ESA/344.

[10] Ward JD, Sutton PC, Werner AD, Costanza R, Mohr SH, Simmons CT (2016) Is Decoupling GDP Growth from Environmental Impact Possible? PLoS ONE 11(10). [View Article] [Google Scholar] 10.1371/journal.pone.0164733 PMC5065220 27741300

[11] European Commission. Communication from the Commission to the European Parliament, the Council, the European Economic and Social Committee and the Committee of the Regions, Next steps for a sustainable European future European action for sustainability 2016; Strasbourg: 739.

[12] International Renewable Energy Agency. Rethinking Energy 2017 Accelerating the global energy transformation 2017.

[13] Andrei JV, Mieila M, Panait M (2017) The impact and determinants of the energy paradigm on economic growth in European Union. PLoS ONE 12(3): e0173282 [View Article] [Google Scholar] 10.1371/journal.pone.0173282 28301505 PMC5354262

[14] World Economic Forum. The Global Competitiveness Report 2014–2015.

[15] Stiglitz J, Amartya S, Fitoussi JP. Report by the Commission on the Measurement of Economic Performance and Social Progress, 2009.

[16] European Commission. Europe 2020 growth strategy, 2010.

[17] OECD. The OECD’s Better Life Index, 2010.

[18] Yale Center for Environmental Law & Policy. Environment Performance Index, 2010–2016.

[19] Global Footprint Network. Ecological Footprint, 1990–2016.

[20] Jorgenson AK, Givens J (2015) The Changing Effect of Economic Development on the Consumption-Based Carbon Intensity of Well-Being, 1990–2008. PLoS ONE 10(5): e0123920 [View Article] [Google Scholar] 10.1371/journal.pone.0123920 25945936 PMC4422519

[21] World Economic Forum. The Europe 2020 Competitiveness Report, Building a more competitive Europe 2014.

[22] Ozturk I. A literature survey on energy-growth nexus. Energy Policy 2010; 38:340–349. [View Article] [Google Scholar]

[23] Payne JE. Survey of the international evidence on the causal relationship between energy consumption and growth. J of Econ. Stud 2010; 37:53–95. [View Article] [Google Scholar]

[24] Tugcu CT, Ozturk I, Aslan A. Renewable and non-renewable energy consumption and economic growth relationship revisited: Evidence from G7 countries. Energy Economics 2012; 34:1942–1950. [View Article] [Google Scholar]

[25] Omri A. An international literature survey on energy-economic growth nexus: Evidence from country specific studies. Renewable and Sustainable Energy 2014; 38:951–959. [View Article] [Google Scholar]

[26] Adewui AO, Awodumi OB. Renewable and non-renewable energy-growth-emissions linkages: Review of emerging trends with policy implications, Renewable and Sustainable Energy Reviews 2017; 69:275–291. [View Article] [Google Scholar]

[27] Chen S, Kuo H, Chen C. The relationship between GDP and electricity consumption in 10 Asian countries. Energy Policy 2007; 35:2611–2621. [View Article] [Google Scholar]

[28] Sadorsky P. Renewable energy consumption, CO2 emissions and oil prices in the G7 countries. Energy Economics 2009a; 31:456–462. [View Article] [Google Scholar]

[29] Sadorsky P. Renewable energy consumption and income in emerging economies. Energy Policy 2009b; 37:4021–4028. [View Article] [Google Scholar]

[30] Apergis N, Payne JE, Menyah K, Wolde-Rufael Y. On the casual Dynamics between emissions, nuclear energy, renewable energy and economic growth, Ecological Economics 2010; 69:2255–2260. [View Article] [Google Scholar]

[31] Apergis N, Payne JE. Renewable energy consumption and economic growth: Evidence from a panel of OECD countries. Energy Policy 2010; 38:656–660. [View Article] [Google Scholar]

[32] Menegaki AN. Growth and renewable energy in Europe: A random effect model with evidence for neutrality hypothesis, Energy Economics 2011; 33:257–263. [View Article] [Google Scholar]

[33] Bayraktutan Y, Yılgör M, Uçak S. Renewable Electricity Generation and Economic Growth: Panel data Analysis for OECD Members. International Research Journal of Finance and Economics 2011; 66:59–67. [View Article] [Google Scholar]

[34] Apergis N, Payne JE. Renewable and non-renewable energy consumption-growth nexus: Evidence from a panel error correction model. Energy Economics 2012; 34:733–738. [View Article] [Google Scholar]

[35] Kazar G, Kazar A. The Renewable energy Production-Economic Development Nexus. International Journal of Energy Economics and Policy 2014; 4(2):312–319. [View Article] [Google Scholar]

[36] Menegaki AN, Ozturk I. Growth and energy nexus in Europe revisited: Evidence from a fixed effects political economy model. Energy Policy 2013; 61: 881–887. [View Article] [Google Scholar]

[37] Chang T, Gupta R, Inglesi-Lotz R, Simo-Kengne B, Smithers D, Trembling A. Renewable energy and growth: Evidence from heterogeneous panel of G7 countries using Granger causality. Renewable and Sustainable Energy Reviews 2015; 52:1405–1412. [View Article] [Google Scholar]

[38] Bildirici M. Relationship between biomass energy and economic growth in transition countries: panel ARDL approach. GCB Bioenergy 2014; 6: 717–726. [View Article] [Google Scholar]

[39] Apergis N, Dănuleţiu DC. Renewable Energy and Economic Growth: Evidence from the Sign of Panel Long-Run Causality. International Journal of Energy Economics and Policy (IJEEP) 2014; 4:578–587. [View Article] [Google Scholar]

[40] Inglesi-Lotz R. The impact of renewable energy consumption to economic growth: A panel data application. Energy Economics 2016; 53(C): 58–63. [View Article] [Google Scholar]

[41] Dogan E, Seker F. The influence of real output, renewable and non-renewable energy, trade and financial development on carbon emissions in the top renewable energy countries. Renewable and Sustainable Energy Reviews 2016; 60:1074–1085. [View Article] [Google Scholar]

[42] Halkos GE, Tzeremes NG. The effect of electricity consumption from renewable sources on countries׳ economic growth levels: Evidence from advanced, emerging and developing economies. Renewable and Sustainable Energy Reviews 2014; 39(C):166–173. [View Article] [Google Scholar]

[43] Sebri M. Use renewables to be cleaner: Meta-analysis of the renewable energy consumption–Economic growth nexus. Renewable and Sustainable Energy Reviews 2015; 42:657–665. [View Article] [Google Scholar]

[44] Saad W, Taleb A. The causal relationship between renewable energy consumption and economic growth: evidence from Europe. Clean Technologies and Environmental Policy 2017; 11:1–10. [View Article] [Google Scholar]

[45] Ito K. CO2 emissions, renewable and non-renewable energy consumption and economic growth: Evidence from panel data for developing countries. International Economics 2017; 151:1–6. [View Article] [Google Scholar]

[46] Afonso TL, Marques AC, Fuinhas JA. Strategies to make renewable energy sources compatible with economic growth. Energy Strategy Reviews 2017; 18:121–126 [View Article] [Google Scholar]

[47] Amri F. Intercourse across economic growth, trade and renewable energy consumption in developing and developed countries. Renewable and Sustainable Energy Reviews 2017; 69:527–534 [View Article] [Google Scholar]

[48] Rio P. Why does the combination of the European Union Emissions Trading Scheme and a renewable energy target makes economic sense?. Renewable and Sustainable Energy Reviews 2017; 74:824–834. [View Article] [Google Scholar]

[49] Alvarez-Herranz A, Balsalobre-Lorente D, Shahbaz M, Cantos JM. Energy innovation and renewable energy consumption in the correction of air pollution levels. Energy Policy 2017; 105:386–397. [View Article] [Google Scholar]

[50] Menegaki AN, Tugcu CT. The sensitivity of growth, conservation, feedback & neutrality hypotheses to sustainability accounting. Energy for Sustainable Development 2016; 34:77–87. [View Article] [Google Scholar]

[51] Menegaki AN, Marques AC, Fuinhas JA. Redefining the energy-growth nexus with an index for sustainable economic welfare in Europe. Energy 2017; 141: 1254–1268 [View Article] [Google Scholar]

[52] Destek MA, Aslan A. Renewable and non-renewable energy consumption and economic growth in emerging economies: Evidence from bootstrap panel causality. Renewable Energy 2017; 111:757–763. [View Article] [Google Scholar]

[53] Alper A, Oguz O. The role of renewable energy consumption in economic growth: Evidence from asymmetric causality. Renewable and Sustainable Energy Reviews 2016; 60:953–959. [View Article] [Google Scholar]

[54] Koçak E, Şarkgüneşi A. The renewable energy and economic growth nexus in black sea and Balkan Countries. Energy Policy 2017; 100:51–57. [View Article] [Google Scholar]

[55] Andrei J, Mieila M, Popescu GH, Nica E, Manole C. The impact and determinants of environmental taxation on economic growth communities in Romania. Energies 2016; 9(11):902 [View Article] [Google Scholar]

[56] Istudor N, Ursăcescu M, Sendroiu C, Radu I. Theoretical framework of organizational intelligence: a managerial approach to promote renewable energy in rural economies. Energies 2016; 9(8):639 [View Article] [Google Scholar]

[57] Becerril H, Rios I. Energy efficiency strategies for ecological greenhouses: experiences from Murcia (Spain). Energies 2016; 9(9): 866 [View Article] [Google Scholar]

[58] Aceleanu M, Șerban C, Pociovălișteanu D, Dimian G. Renewable energy: A way for a sustainable development in Romania. Energy Sources, Part B: Economics, Planning, and Policy 2017; 12(11): 958–963.

[59] Hartmann B, Börcsök E, Groma VA, Osán J, Talamon A, Török S, et al Multi-criteria revision of the Hungarian Renewable Energy Utilization Action Plan–Review of the aspect of economy. Renewable and Sustainable Energy Reviews 2017; 80:1187–1200. [View Article] [Google Scholar]

[60] Nikolaev A, Konidari P. Development and assessment of renewable energy policy scenarios by 2030 for Bulgaria, Renewable Energy 2017; 111:792–802. [View Article] [Google Scholar]

[61] Furuoka F. Renewable electricity consumption and economic development: New findings from the Baltic countries. Renewable and Sustainable Energy Reviews 2017; 71:450–463. [View Article] [Google Scholar]

[62] European Environment Agency. Renewable energy in Europe 2016. Recent growth and knock-on effects 2016.

[63] The Romanian Government. National Reform Plan 2016 Bucharest.

[64] Eurostat. Renewable energy statistics / Statistics Explained 2016.

[65] European Commission. Clean Energy For All Europeans. Communication from the Commission to the European Parliament, the Council, the European Economic and Social Committee, the Committee of the Regions and the European Investment Bank 2016.

[66] European Environment Agency. Renewable Renewable energy in Europe 2017. Recent growth and knock-on effects 2017.

[67] Pesaran MH, Pesaran B. Working with Microfit 4.0: Interactive Econometric Analysis, Oxford University Press, Oxford; 1997.

[68] Pesaran MH, Shin Y. An Autoregressive Distributed Lag Modelling Approach to Cointegration Analysis. In S. Strom, (ed) Econometrics and Economic Theory in the 20th Century: The Ragnar Frisch Centennial Symposium, Cambridge University Press, Cambridge; 1999.

[69] Pesaran HM, Shin Y, Smith RP. Pooled mean group estimation of dynamic heterogeneous panels. Journal of the American Statistical Association 1999; 94:621–634. [View Article] [Google Scholar]

[70] Pedroni P. Critical Values for Cointegration tests in Heterogeneous Panels with Multiple Regressors, Oxford Bulletin of Economics and Statistics 1999; 61:653–670. [View Article] [Google Scholar]

[71] Kao C. Spurious regression and residual-based tests for cointegration in panel data. Journal of Econometrics 1999; 90:1–44. [View Article] [Google Scholar]

[72] Levin A, Lin F, Chu C. Unit Root Tests in Panel Data: Asymptotic and Finite-Sample Properties, Journal of Econometrics 2002; 108:1–24. [View Article] [Google Scholar]

[73] Breitung J. The Local Power of Some Unit Root Tests for Panel Data In Nonstationary Panels, Panel Cointegration, and Dynamic Panels, Advances in Econometrics, Baltagi B. (ed.), Amsterdam, 2000; 15:161–178.

[74] Im KS, Pesaran MH, Shin Y. Testing for unit roots in heterogeneous panels, Journal of Econometrics 2003; 115:53–74. [View Article] [Google Scholar]

[75] Maddala GS, Wu SA. Comparative Study of Unit Root Tests with Panel Data and a New Simple Test. Oxford Bulletin of Economics and Statistics 1999; 61:631–652. [View Article] [Google Scholar]

[76] Johansen S. Statistical analysis of cointegration vectors. Journal of Economic Dynamics and Control 1988; 12:231–254. [View Article] [Google Scholar]

[77] Fisher RA. Statistical Methods for Research Workers, 4th Edition; Oliver and Boyd: Edinburgh; 1932.

[78] Dogan E. The relationship between economic growth and electricity consumption from renewable and non-renewable sources: a study of Turkey. Renewable and Sustainable Energy Reviews 2015; 52: 534–546. [View Article] [Google Scholar]

[79] Armeanu D, Vintila G, Gherghina S. Does Renewable Energy Drive Sustainable Economic Growth? Multivariate Panel Data Evidence for EU-28 Countries. Energies 2017; 10(3): 381. [View Article] [Google Scholar]

[80] European Commission. A policy framework for climate and energy in the period from 2020 up to 2030. Brussels 2014.

[81] Letta E, Pellerin-Carlin Th, Fernandes S, Rubio E, Vinois JA. Making the Energy Transition a European Success. Report—Jacques Delors 2017; 114:100–145.

